# Poverty, sanitation, and *Leptospira* transmission pathways in residents from four Brazilian slums

**DOI:** 10.1371/journal.pntd.0009256

**Published:** 2021-03-31

**Authors:** Hussein Khalil, Roberta Santana, Daiana de Oliveira, Fabiana Palma, Ricardo Lustosa, Max T. Eyre, Ticiana Carvalho-Pereira, Mitermayer G. Reis, Albert I. Ko, Peter J. Diggle, Yeimi Alzate Lopez, Mike Begon, Federico Costa

**Affiliations:** 1 Institute of Collective Health, Federal University of Bahia, Salvador, Brazil; 2 Swedish University of Agricultural Sciences, Umeå, Sweden; 3 Instituto Gonçalo Moniz, Fundação Oswaldo Cruz, Ministério da Saúde, Salvador, Brazil; 4 Centre for Health Informatics, Computing, and Statistics, Lancaster University Medical School, Lancaster, United Kingdom; 5 Faculdade de Medicina da Bahia, Federal University of Bahia, Salvador, Brazil; 6 Department of Epidemiology of Microbial Diseases, Yale School of Public Health, New Haven, United States of America; 7 Department of Evolution, Ecology and Behaviour, The University of Liverpool, United Kingdom; Universidade Federal de Pelotas, BRAZIL

## Abstract

Residents of urban slums suffer from a high burden of zoonotic diseases due to individual, socioeconomic, and environmental factors. We conducted a cross-sectional sero-survey in four urban slums in Salvador, Brazil, to characterize how poverty and sanitation contribute to the transmission of rat-borne leptospirosis. Sero-prevalence in the 1,318 participants ranged between 10.0 and 13.3%. We found that contact with environmental sources of contamination, rather than presence of rat reservoirs, is what leads to higher risk for residents living in areas with inadequate sanitation. Further, poorer residents may be exposed away from the household, and ongoing governmental interventions were not associated with lower transmission risk. Residents at higher risk were aware of their vulnerability, and their efforts improved the physical environment near their household, but did not reduce their infection chances. This study highlights the importance of understanding the socioeconomic and environmental determinants of risk, which ought to guide intervention efforts.

## Author summary

Globally, more than 1 billion residents live in urban slums, where inadequate sanitation increases the spread of pathogens and their animal hosts. Leptospirosis is a globally distributed bacterial disease, and in urban areas the bacteria is spread by brown rats. Humans become infected upon exposure to water or soil/mud that was contaminated with the urine of infected rats. In four urban slums in the city of Salvador, Brazil, we used a range of individual, socioeconomic, and environmental variables to understand how they influence to one another and define o leptospirosis exposure in residents. Poverty and sanitation influenced the degree to which residents were exposed to sewer water and mud. Further, we found that while residents tried to improve the physical environment near their household, living in an area with poor sanitation mean that their infection risk remained high. This work highlight the importance of socioeconomic and environmental factors in determining disease risk, and suggests that governmental interventions should focus on improving sanitation changes in the poorest parts of the community.

## Introduction

More than 50% of the world population lives in urban areas, and the fastest growing cities are in low- and middle-income countries (LMICs)[1]. In many regions, the majority of urban residents live in slums[1]: high-density, low-income communities with limited infrastructure and minimal access to healthcare and other essential services[2]. Because of poor sanitation and inadequate waste management, slum residents suffer a higher burden of infectious diseases than the rest of the city, including dengue fever[3] and severe leptospirosis[4]. Yet, even within communities that may appear uniformly poor, there are gradients in ill health that reflect fine-scale social, economic and physical conditions[5,6]. The literature on socioeconomic inequalities in health is extensive for chronic diseases, especially from high-income countries, but for neglected tropical diseases, there is limited evidence for unequal distribution of risk based on socioeconomic and physical conditions[7]. Here we seek to understand the direct and indirect pathways that drive the variation in leptospirosis transmission in slum communities in Salvador, Brazil.

Encouragingly, studies on the control of infectious diseases, especially diseases of poverty, have started to adopt a broader view of risk and vulnerability[8]. For example, incorporating local knowledge and perceptions, and measures of socioeconomic and environmental conditions, partially explained spatial heterogeneity in dengue fever risk in Colombia[9]; while in rural Kenya, household socioeconomic status explained differences in Malaria and hookworm infections among low-income households, potentially due to unequal access to sanitation and protective equipment[5].

In Brazil, the urban poor suffer from multiple vector-borne and zoonotic infections, including leptospirosis, a major disease of poverty. Worldwide, leptospirosis causes around 1 million human infections and approximately sixty thousand deaths annually[10]. Severe forms of the disease, such as Weil’s diseases or severe pulmonary haemorrhagic syndrome, have high mortality rates (10% and 50%, respectively[11]). Infection is caused by pathogenic bacteria of the genus *Leptospira*. While many mammalian and reptilian species are reservoirs for the bacteria [12]in urban settings it is predominantly carried and transmitted by the Norway rat (*Rattus norvegicus*)[13]. Humans contract leptospirosis upon contact with soil and water contaminated with rat urine. The majority of leptospirosis cases reported annually in Brazil occur in urban slums[4], where trash and open sewers support high densities of rats and expose humans to sources of contamination[6,14]. Governmental interventions to reduce reservoir abundance, namely trash collection and application of rodenticides by the Center for Control of Zoonoses (CCZ), are absent in many parts of the communities, and where present, their effectiveness has rarely been evaluated[15].

In Salvador city in the northeast of Brazil, long-term cohort[6], cross-sectional[16], and case-control[17,18] studies, have identified demographic, environmental, and socioeconomic risk factors of leptospirosis. These studies found that risk increased with rat sightings[18], with household proximity to open sewers[17] and accumulated trash[16], and with contact with sources of contamination such as mud[6]. Risk was higher in lower income households, for males, and for illiterate residents[6]. The majority of residents were, though, aware of the disease, its reservoir and transmission routes, and the protective measures that may help avoid infection[19].

In spite of this knowledge of risk factors for urban leptospirosis transmission, it has been a challenge to develop a framework that provides a structured, hierarchical understanding of the relationship among individual, socioeconomic, and physical dimensions of risk. The framework would help us to simultaneously account for and understand the relationships among risk factors, and the pathways through which they drive transmission. This applies equally to other diseases of poverty, especially in LMICs. Such understanding is necessary for the design and implementation of enlightened intervention strategies where they would be effective.

Here, therefore, our aim is understand the context of urban leptospirosis transmission on a community scale, using a large number of individual, socioeconomic, and environmental variables, including existing interventions, to separate direct and indirect determinants of risk. Based on two decades of research on urban leptospirosis, we formulated and tested a structural equation model (SEM) for *Leptospira* transmission in four previously unstudied urban slums in the city of Salvador. We were especially interested in understanding the differences in risk stemming from fine-scale gradients in socioeconomic and physical conditions within each community. The framework is based on the factors associated with leptospirosis incidence and sero-prevalence that were established in previous studies. Within this framework, we hypothesized pathways that linked risk factors to each other and, where relevant, directly to resident sero-positivity. We believe that such a framework, resolved at a within-community scale, will be useful for stakeholders, especially local authorities, for addressing and ultimately interrupting the most relevant pathways of transmission.

## Materials and methods

### Ethics statement

The Research Ethics Committee of the Institute of Collective Health (UFBA) approved blood collection, applying the questionnaire, and laboratory analysis of human sera (permit 041/17-2.245.914.17–2.245.914).

### Sero-survey in the four communities

**Fig 1:**
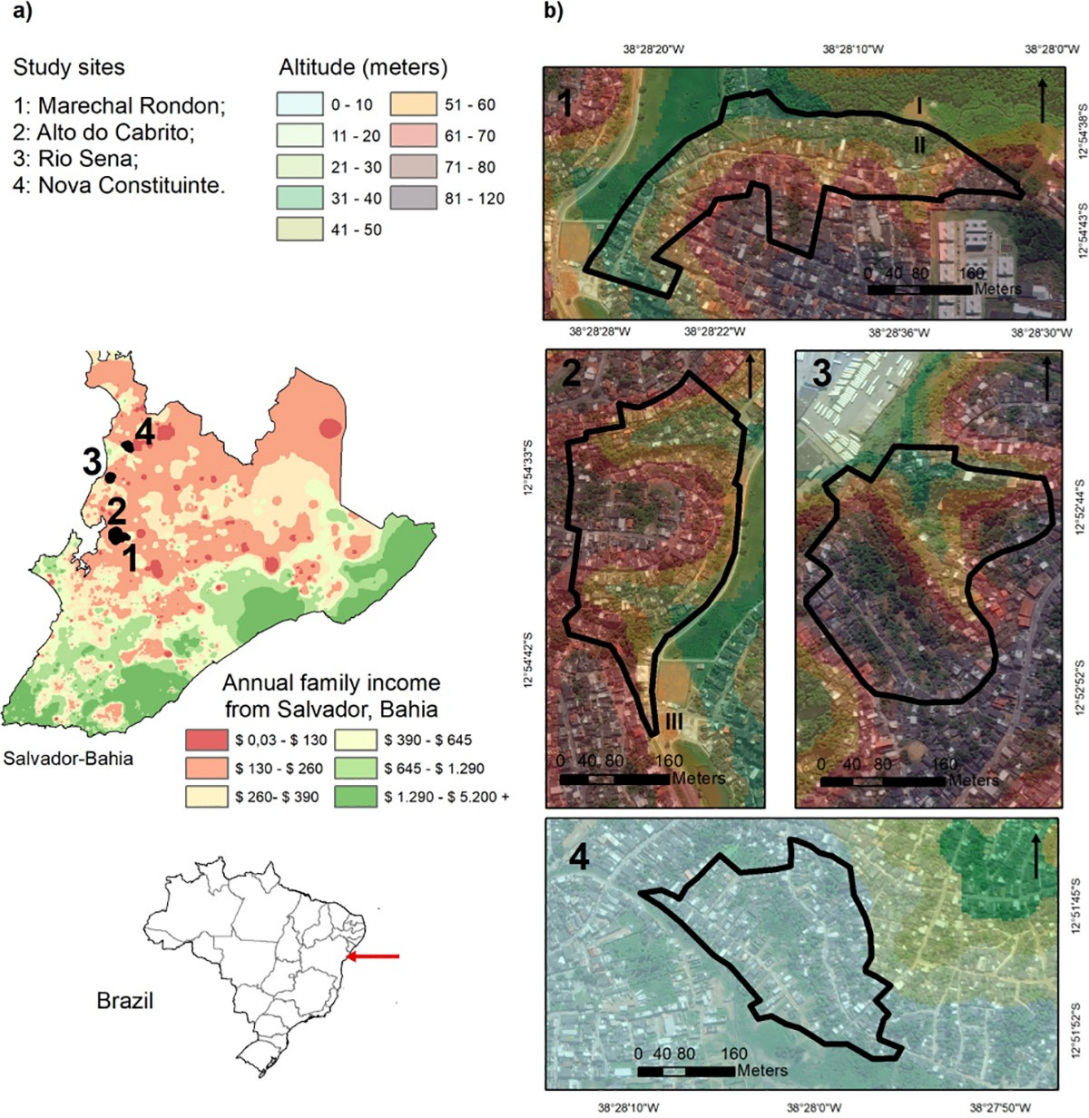
**Map of the four study areas in the city of Salvador:** a) showing the location of the communities within the city, annual income distribution in the city, b) altitude gradient within each community.

We conducted a cross-sectional sero-survey in four slum communities in the north-western periphery of the city of Salvador[20]. The communities are Alto do Cabrito (AdC), Marechal Rondon (MR), Nova Constituinte (NC), and Rio Sena (RS), all low-income informal settlements with generally poor sanitation and infrastructure (Fig 1) [20]. Incidence of severe hospitalized leptospirosis in urban slums in Salvador is *ca*.19.8 cases per 100,000 inhabitants[14].

In each community, the study area ranged between 0.07 and 0.09 km^2^. The study areas were selected to maximize income and altitudinal gradients, while considering accessibility and risk for participants and researchers (Fig 1, household income data from extracted from [20]). The socioeconomic and environmental differences within each community are greater than the differences among communities (see Table A in S1 File for summaries of all variables *per* community). Nevertheless, MR, AdC, and RS have a clear altitudinal gradient (Fig 1), but the community of NC is largely flat, with standing water in the central part of the study area–where lower income households tended to be located. In all communities, there were areas with open sewers, unpaved roads, and households without easy access to the main roads, but the largest open sewer is at the bottom of the valley in RS (north-eastern part of the study area), where only 55% of households have paved access, which was the lowest among the four study areas.

Sample size calculations, considering leptospirosis prevalence similar to that found in a previous study in Salvador (14.3%) [16], indicated that between 300 and 350 residents in each community were required to reach a precision of 0.05 and confidence level of 0.95.

We first conducted a census of each site and identified all households. We systematically visited each householdand contacted inhabitants who met the eligibility criteria for the study, namely, individuals ≥5 years of age who slept ≥3 nights per week in a household. Enrolled residents provided written informed consent[6,14]; trained phlebotomists then collected blood samples and administered questionnaires. All individual data were anonymised. Data collection was between March and October 2018.

The sero-survey questionnaire was a modified version of the questionnaire used in the Pau da Lima community (see Table B in S1 File) [6,18]. In addition to data on demography, income, and other socioeconomic indicators, we collected data on domestic and peri-domestic environmental factors such as presence of open sewers, flooding, trash accumulation, and rat sightings. We also asked residents about exposure to sources of contamination such as mud, sewage, and floodwater in the past 12 months. Residents informed us about their perceived vulnerability to leptospirosis, if they utilized protective measures to reduce rat abundance and potential exposure, whether there was trash collection in their street, and if CCZ agents had applied rodenticides peri-domestically in the past six months (see Table C in S1 File for full list of variables used in the study).

To identify previous exposure to *Leptospira*, we performed microscopic agglutination tests (MAT)[16] on sera obtained from the human blood samples and determined titers of agglutinating antibodies. We used a panel of five reference strains (WHO Collaborative Laboratory for Leptospirosis, Royal Tropical Institute, Holland) and two clinical isolates (see Reis et al.[16] for more details). We screened with serum with dilutions of 1:25, 1:50 and 1:100. When agglutination was observed at a dilution of 1:100[21], the sample was titrated in serial two fold dilutions to determine the highest positive titer.

### Statistical analysis

Our framework aimed to understand the relationship among risk factors and identify the pathways governing the transmission of leptospirosis in urban slums. We evaluated this framework using data collected from the four communities through structural equation modelling (SEM). SEM is a multivariate technique that combines confirmatory factor analysis and multiple regression to evaluate whether the data support a pre-defined hypothetical model. SEM hypothesizes causal relationships among variables and tests them through linear equations. The models can include continuous or binomial variables that are measured, latent, or both. Latent variables are variables that represent a theoretical construct that cannot be directly measured, but is instead inferred from measured variables. The standardized values assigned by the model to the “pathways” between variables are estimates of the strength of that relationship (based on the observed covariance matrix) after taking into account other relationships specified in the model[22].

After fitting a hypothetical model, which included all pathways supported by prior studies[4,6,14,16–18], we removed non-significant pathways (*p* > 0.1, shown in D in supplementary information). To test the overall fit of the model, we used RMSEA (root mean squared error of approximation) and SRMSR (standardized root mean squared residual), rather than χ^2^. χ^2^ is appropriate for models with a sample size in the range of n = 75–200, but for n > 400 it will often be significant, i.e. indicating bad fit^23^. RMSEA is based on χ^2^ but is standardized by degrees of freedom. An RMSEA value of < 0.05 indicates a good fit. SRMSR is an absolute measure of fit, defined as the standardized difference between the observed correlation and the predicted correlation, for which a value of < 0.08 is considered a good fit[23].

**Fig 2:**
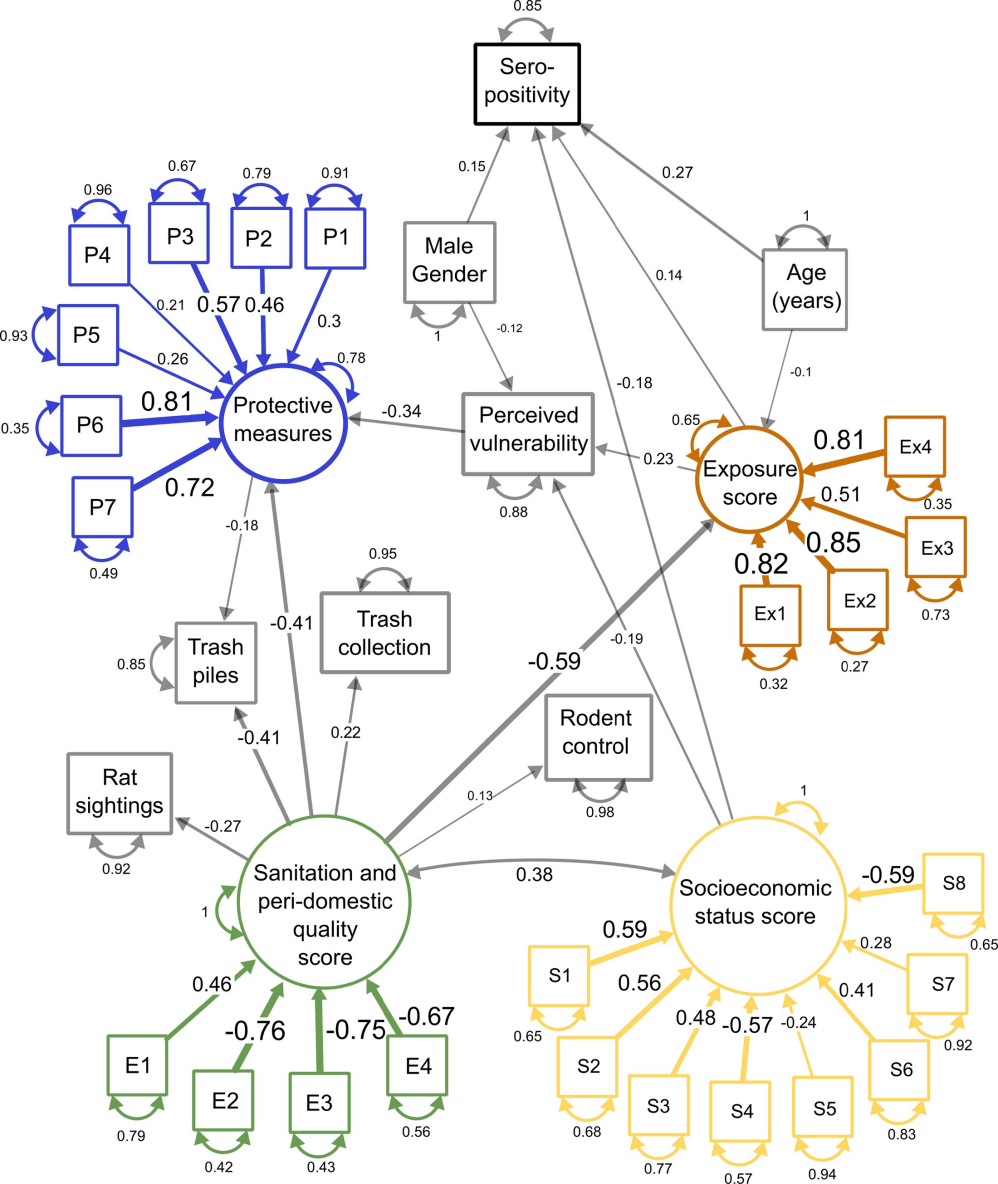
Final Structural equation model linking socioeconomic, environmental, exposure and demographic factors to leptospirosis sero-positivity, while accounting for the relationships among these factors. There are 31 measured variables (in boxes) in the model (including sero-positivity), 23 of those representing 4 latent variables (in circles). Unless otherwise specified, the questions had binary responses with reference as “no”. Sanitation and peri-domestic quality score: E1 = Access to household is paved, E2 = Household floods after rain, E3 = Sewer floods, E4 = Presence of open sewer within 10 meters of the household. Socioeconomic status score: S1 = Owns a car, S2 = Owns a computer, S3 = Owns a washing machine, S4 = Food runs out before resident can afford to restock. S5 = Household ownership (reference: owned, rented), S6 = Education level (>5 years, 5–9 years, 9–12 years, >12 years of education). S7 = Maximum income in the household (in Brazilian Reais, continuous), S8 = Household has unplastered walls. Exposure score: In the past 12 months, the resident had Ex1 = Contact with sewage water, Ex2 = Contact with floodwater, Ex3 = Cleaned a sewer, Ex4 = Contact with mud. Protective measures: In the household P1 = Prevent trash accumulation, P2 = Wear shoes, P3 = Kill rats, Outside the household P4 = Clean peri-domestic area, P5 = Wear boots or gloves, P6 = Kill rats, P7 = Restructure sewer flow. One-sided arrows represents a causal influence originating from the variable at the base of the arrow, with the width of the arrow representing the strength of the relationship. Double-sided arrows represent a correlation. The small double-sided arrows and numbers next to each variable represent the error variance.

The hypothetical SEM model included both directly measured and latent variables. The latent variables were *socioeconomic status* (inferred, for example, by education, maximum income in the household, owning a car), *sanitation and peri-domestic quality* (e.g. flooding frequency, presence of open sewer), *individual exposure to sources of contamination* (e.g. walking in mud or sewer water), and *individual preventative measures* (e.g. cleaning, preventing trash from accumulating in the household, applying rodenticides). The list of measured variables contributing to the latent variables is presented in Fig 2 and the description of all variables is in Table C in S1 File. Directly measured variables in the SEM were age, gender, perceived vulnerability to leptospirosis, rat sightings, trash accumulation, presence of a trash collection service, rodenticide application by CCZ, and sero-status for leptospirosis.

We were unable to include household and community identities as variables in SEM due to computational issues, as we only enrolled one to three residents per household in 70% of households in each community (range of number of participants *per* was 1–10). Thus, to confirm SEM results and account for community effects and the correlation among multiple observations from the same household, we fitted a generalized linear mixed effects model (GLMM) with a binomial error distribution. The response variable was sero-postivity and the candidate predictor variables in the GLMM were the variables maintained in the final SEM plus community, and we included household identity as a random effect. Using the variables maintained in the final SEM, we fitted and compared subset of models comprising all possible combinations of variables. The comparisons were based on Akaike Information Criteria (AIC)[24] and the top five candidate models, including the final model with the lowest AIC, are presented in Table E in S1 File. To further illustrate how the chain of transmission implied by SEM originates in poor socioeconomic conditions, we fitted three additional GLMMs with a binomial error distribution. The first model tested the association between the latent variable *socioeconomic status* and exposure to a) sewer water in the past 12 months, and then second model between b) exposure to sewer water and perceived vulnerability to leptospirosis, and finally, c) exposure to sewer water and sero-status for leptospirosis. In the three models, we included resident age, gender, and community as fixed effects, and household identity as a random effect. We compared the AIC of each model with that of a null model.

All analyses were performed in R statistical software[25], using lavaan[26], tidyverse[27], and lme4[28] packages.

## Results

In the four communities, we identified 2,749 eligible residentsthrough household visits or through other members of the household. Of the 2749 residents identified, 1,807 (66%) of these were successfully contacted, and 1,318 (73% of contacted) subsequently enrolled in the study: 338 in MR, 375 in AdC, 306 in NC, and 299 in RS. There was a higher proportion of female participants in all communities, ranging from 56.5% in AdC and RS to 59.5% in MR. This gender bias was not different among the four communities (χ^2^ test, df = 3, *p* = 0.84). Unadjusted *Leptospira* sero-prevalence (% of positive residents, CI calculated using the Clopper–Pearson method [29]) was 12% in MR (95% CI 8.8–16.5), 11.3% in AdC (8.1–15.1), 10% in NC (6.8–14.2), and 13.3% in RS (9.4–18.0). In total, 91% of the 1,318 enrolled residents were aware of leptospirosis. However, for 321 individuals, there was missing information for socioeconomic variables only the head of the household, not always contactable, could provide. These residents were younger than the overall average (21.2 and 34.3 years, respectively, t-value = -12.85, df = 558, *p* < 0.01), but gender composition did not differ (59.5% and 57.4% females, respectively, χ^2^ = 0.28, df = 1, *p* = 0.59). Hence, ultimately, 997 residents from 496 households were included in SEM and subsequent GLMMs. Table 1 summarises other key demographic, socioeconomic, and environmental variables in the four communities.

**Table 1:** Key variables measured in the four communities.

	Marechal Rondon	Alto do Cabrito	Nova Constituinte	Rio Sena	Four Communities
Age	Mean (SD)	Mean (SD)	Mean (SD)	Mean (SD)	Mean (SD)
	39 (19.7)	35.3 (17.7)	32.7 (17.7)	29.7 (17.5)	34.3 (18.5)
Gender	Female %	Female %	Female %	Female %	Female %
	59.5	56.5	56.9	56.5	57.4
Maximum family income ($R)	Mean (SD)	Mean (SD)	Mean (SD)	Mean (SD)	Mean (SD)
	771 (737)	833 (663)	731 (913)	642 (571)	750 (731)
Open sewer near household	% Yes	% Yes	% Yes	% Yes	% Yes
	39.6	20.3	32	39.5	32.3
Resident had contact with sewer in the last 12 months	% Yes	% Yes	% Yes	% Yes	% Yes
	29.6	13.3	18.6	23.1	20.9
Trash collection in the street	% Yes	% Yes	% Yes	% Yes	% Yes
	80.5	72	100	81.9	82.9
Perceived vulnerability to Letpospirosis	% Yes	% Yes	% Yes	% Yes	% Yes
	65.1	54.1	63.4	58.9	60.2

### SEM

After removing non-significant pathways, the final SEM model had 55 free parameters and 522.5 degrees of freedom. The model fit, based on a RMSEA value of 0.038 (90% CI 0.036–0.045) was good. SRMR of 0.091 was only slightly higher than the 0.08 cut-off for a good fit. Taking these values together, we proceeded with interpretation without further adjustment in model pathways. All reported values for pathways are standardized coefficients to facilitate comparisons among relationships at different levels.

The focal response variable in the framework was sero-positivity to *Leptospira*. This decreased as *socioeconomic status* of a resident increased (standardized estimate = -0.18 [-0.32, -0.04, 95% CI]), increased with *exposure to sources of contamination* (+0.14 [0.01, 0.26]), and with age (+0.27 [0.18, 0.37]; expected given the cumulative nature of sero-positivity[16,30]), and was higher in males than in females (+0.15 [0.05, 0.25]). Equally notable was the absence of a direct effect on sero-positivity of the latent variable *sanitation and peri-domestic quality* and measured variables accumulated trash near the household and rat sightings (Fig 2 and Table C in S1 File).

The GLMM model for sero-positivity, fitted to account for nestedness in the data, confirmed these results. The model with all variables maintained the final SEM were selected in the final model (ΔAIC > 2) (Table 2). The risk of being seropositive decreased as *socioeconomic status* increased (OR = 0.28 [0.15, 0.53, 95% CI), and increased with *exposure to sources of contamination* (1.9 [1.26, 2.86]), and with age in years (1.04 [1.02–1.05]). Males were twice as likely to be positive compared to females (2.0 [1.27–3.15]). Sero-prevalence was also marginally higher in RS (1.84 [0.92–3.68], *p* = 0.087), compared to MR, AdC, and NC.

**Table 2:** Summary of generalized linear mixed effects model with sero-status of residents as binary response variable. The predictor variables were those maintained in the final structural equation model, but also controls for community and household (random effect) effects.

	Sero-status		
*Predictor*	*Odds Ratio*	*95% CI*	*p*
Intercept (Marechal Rondon)	0.01	0.00–0.03	**<0.001**
**Community**			
Alto do Cabrito	1.61	0.81–3.22	0.176
Nova Constituinte	1.66	0.81–3.42	0.166
Rio Sena	1.84	0.94–3.68	0.087
Socioeconomic score	0.28	0.15–0.53	**<0.001**
Exposure score	1.88	1.26–2.86	**0.003**
Sex	2.00	1.27–3.15	**0.003**
Age	1.04	1.02–1.05	**<0.001**
**Random Effects (household)**			
σ^2^	3.29		
τ_00 household_	1.03		
ICC	0.24		
N _household_	495		
Observations	997		

The latent variables representing *sanitation and peri-domestic quality* and *socioeconomic status* (see SI for contributing variables) directly or indirectly influenced all other response variables. They were also correlated (+0.38 [0.28, 0.48], Fig 2). Whereas, as noted above, the effect of poverty on sero-positivity was not captured by other (indirect) pathways in the model, sanitation influenced risk through exposure. Residents living in areas with poor sanitation were more likely to be exposed to sources of contamination (-0.59 [-0.66, -0.51]), and through exposure, were ultimately more likely to be seropositive (+0.14).

Socioeconomic status also directly influenced perceived vulnerability to leptospirosis. As socioeconomic status increased, there was a decrease in perceived vulnerability (-0.19 [-0.30, -0.08]). Those experiencing greater exposure to risk also perceived themselves to be more vulnerable (+0.23). However, men were less likely than women to perceive themselves as vulnerable to leptospirosis (-0.12 [-0.20, -0.04]) despite having higher sero-prevalence.

Residents living in areas with inadequate sanitation were more likely to take protective measures to avoid contamination (-0.41[-0.54, -0.27]), and these efforts were associated with lower trash accumulation (-0.18 [-0.30, -0.05]), but they were not associated with either exposure or sero-positivity (Fig D in S1 File). Governmental interventions (trash collection, +0.22 [0.11, 0.33]) and rodenticide application by CCZ (+0.13 [0.02, 0.23]), were less likely to reach areas with poor sanitation. This was supported by the finding that residents living in these areas were more likely to have trash accumulated near their house (-0.41[-0.52, -0.30]) and to see rats (-0.27 [-0.37, -0.17]).

**Fig 3:**
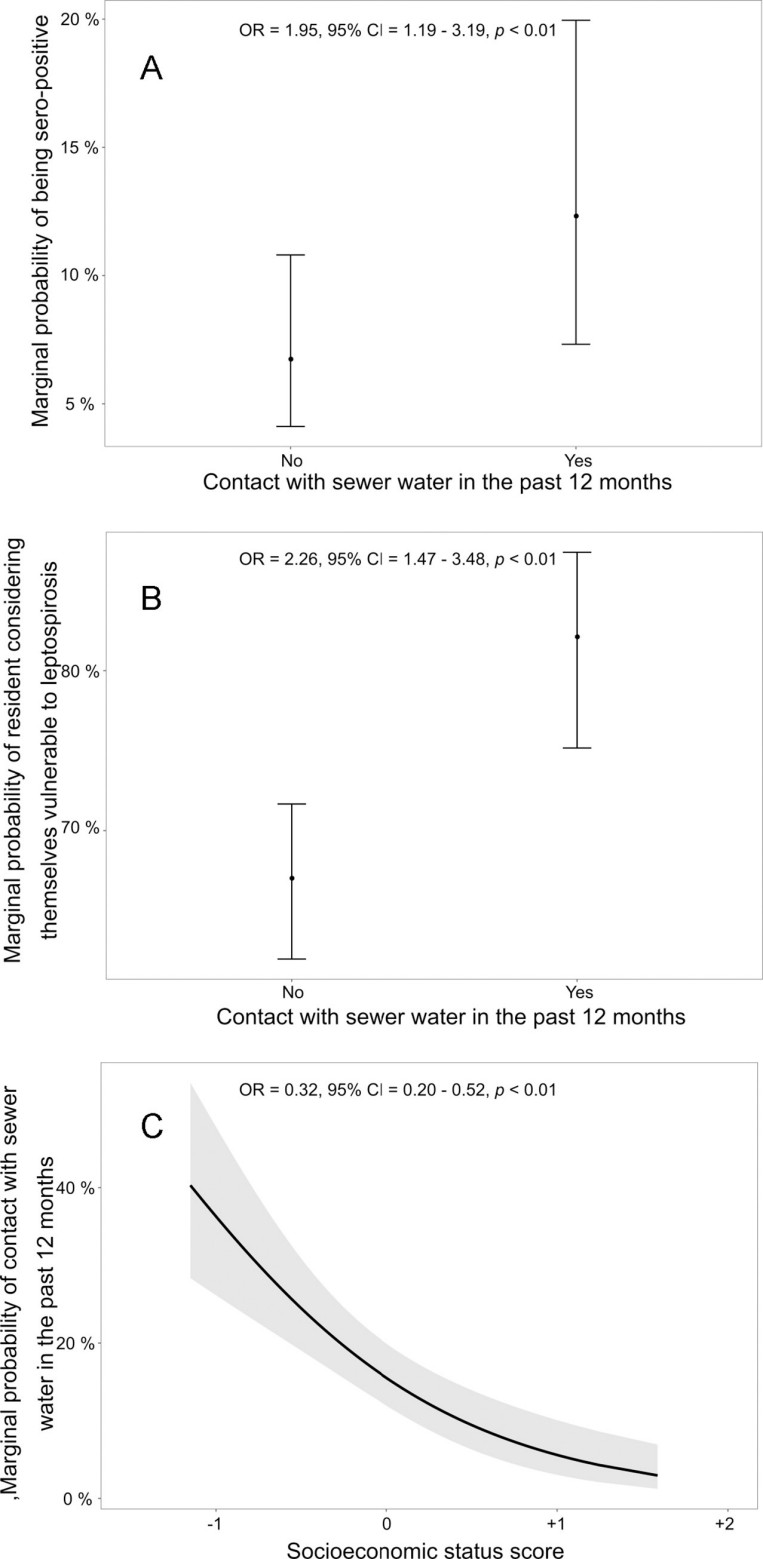
Model predicted relationships of three generalized linear mixed effect models illustrating the relationship between inadequate sanitation, poor socioeconomic conditions, and leptospirosis risk. The panels show predicted marginal probability of a) sero-positivity in relation to contact with sewer water, b) perceived vulnerability to leptospirosis in relation to contact with sewer water, and c) contact with sewer water as a function of socioeconomic status (score).

The three additional GLMMs further showed how low socioeconomic status and exposure to contamination drive transmission. Residents of lower *socioeconomic status* were more likely to have had contact with sewer water in the last 12 months (OR = 0.32 [0.20, 0.52, 95% CI]), and in turn, contact with sewer water almost doubled the odds of being seropositive (1.95 [1.19, 3.19]) (Fig 3). Also, residents who had had contact with sewer water were more likely to perceive themselves vulnerable to leptospirosis (2.26 [1.47, 3.48]). All three models (with one predictor variable) had lower AIC (ΔAIC > 2) compared to the null model with only an intercept and random effects.

## Discussion

We considered a range of individual, socioeconomic, and environmental variables to identify direct and indirect pathways of leptospirosis transmission in four urban slum communities in Salvador, Brazil. Low socioeconomic status and inadequate sanitation were the key variables in the system, influencing exposure to sources of contamination, rat infestation and trash accumulation near households, access to services by residents, and ultimately individual *Leptospira* sero-positivity. But crucially, whereas within each community poverty appeared directly associated with *Leptospira* seropositivity in our model, individual exposure to sources of contamination was the pathway through which inadequate sanitation affected transmission–that is, inadequate sanitation, as such, had indirect effects.

Inadequate sanitation and infrastructure has been associated previously with leptospirosis[16] and other rodent-borne diseases such as Lassa fever[31]. For example, Hagan et al.[6], in a four-year cohort study in Salvador reported proximity to accumulated trash and rat sightings as risk factors for leptospirosis (see also Fig D in S1 File). Chikungunya infection in Salvador is also higher in households without paved access [32]. Here, though, neither of these factors was related to exposure and sero-prevalence in the SEM, but were themselves driven by sanitation and physical environment near the household. Thus, our results and the exposure profile that we have formulated, suggest that it is inadequate sanitation, not rat sightings and trash *per se*, that directly promotes exposure to sources of contamination (Fig 2). Indeed, previous work in Pau da Lima slum in Salvador found that repeated exposure to *leptospira* is associated with living close to open sewers, and suggested that infrastructural interventions would be necessary to reduce risk.

It is noteworthy too that we detected direct effects of poverty on *Leptospira* sero-positivity that were not acting through other variables such as increased exposure. Future work, therefore, should seek factors beyond those studied here in seeking to understand why, even within these marginalised communities, the most marginalised are especially vulnerable to infectious disease.

Alongside low socioeconomic status, men, too, had higher sero-prevalence, in line with previous studies on leptospirosis[6,14]. However, again, for male gender, the pathway through which risk is increased remains unidentified. In our model, men were less likely to perceive themselves vulnerable to infection compared to women. However, perceived vulnerability to leptospirosis was not associated with higher sero-prevalence. Hence, we tentatively rule out that men had higher sero-prevalence because they underestimated risk.

Rather, we hypothesize that the risk suffered by residents of lower socioeconomic status in general and men in particular, irrespective of where they live and of contact with mud and dirty water, stems from their movement patterns and activities they undertake. In Pau da Lima, men visited a larger area compared to women[33], and it is likely that residents of lower socioeconomic status live far from transportation networks, increasing their contact with *Leptospira* away from the household. To test these hypotheses, future studies ought to investigate how movement and associated exposure away from the household vary with socioeconomic status and gender.

Protective measures taken by residents to did not appear to reduce rat sightings or leptospirosis risk, though they were successful in reducing trash accumulation. Further, trash collection and rodent control services, where available, did not mitigate transmission risk. Rodenticide campaigns have scarcely been evaluated in urban slums, but they appear to be less effective here[15] compared to non-slum areas[34]. Similarly, efforts to control to Dengue fever risk through controlling *Aedes* mosquito populations has had limited success, due to the continuous financial and logistic investment required for interventions, which include spraying insecticides and removing mosquito breeding habitats [35]. Our results suggest that the focus of interventions to reduce leptospirosis should be instead on improving infrastructure and sanitation in the poorest parts of the community. If successful, this would reduce rat infestation and resident exposure to mud and dirty water, while better infrastructure is also likely to enable residents to access the main road or transportation without increased exposure to environmental contamination.

The challenges related to leptospirosis transmission in Salvador reflect issues of infrastructure deficiency and marginalization, a finding which extends to other urban slums and other diseases in LMICs[36]. Residents of urban slums are often excluded from opportunities and services that would enable them to improve their life and health[36]. For example, unpaved, low quality and narrow roads, and frequent flooding are likely to make access for trash collection trucks difficult[37], further isolating these areas. Rodent control efforts, constrained by limited budgets, have tended to focus on more affluent parts of the city of Salvador[36]. Similarly, dengue transmission in the city of Fortaleza, Brazil, was also associated with poverty and lack of services. There, violence interfered with vector-control efforts, which led to higher incidence[38].

Teasing apart direct and indirect drivers of transmission revealed the context within which transmission occurs and the relevant pathways governing it., This facilitates defining intervention targets, assessing their effectiveness[39], and prioritizing their implementation in the most vulnerable parts of the community. Indeed, while rodent control through rodenticide application does not appear to be effective (this study and [15]), socioeconomic[40] and sanitary [41]interventions have improved health outcomes for the urban poor in Brazil. Further, community-based approaches that involve residents and local stakeholders in designing and implementing risk-reduction interventions [42], have reduced malaria [43]and diarrheal disease risk in urban areas. Also, the control of mosquito vectors in low-income areas improved substantially when local residents were involved from the start[44]. For leptospirosis, the effectiveness of e.g. rat control efforts may also increase if interventions become community based. To inform these efforts, we still need to evaluate how different combinations of environmental improvements affect rat infestation [45].

Nevertheless, in a serological study such as ours, self-reported data (e.g. exposure to mud) and infections acquired earlier are likely to have amplified and/or weakened connections among variables in the data, the clearest example of which is the strong association between age and sero-positivity. Although earlier exposure likely exaggerated the strength of the association between age and sero-positivity, older residents may have participated in activities that increased their exposure to sources of contamination, e.g. cleaning canals or sewers.

More generally, our framework has sought to contextualize individual risk at a fine scale, within individual slum communities, and thus serve as a starting point for devising effective, sustainable strategies for combating vector borne and zoonotic diseases in urban slums in Brazil and other LMICs.

## Supporting information

S1 File Table A. Hypothesized relationships in the structural equation model (**a)** kept in the final model and **b)** that were removed (*p* > 0.1). The relationships that were removed included the effects of trash collection on rat sightings and trash accumulation, the relationship between sanitation and peri-domestic quality score and sero-positivity, and socioeconomic status score and exposure. The numbers next to the paths refer to the studies, listed below, that reported the hypothesized relationships. **Table B.** Full list of questions in the sero-survey applied in the four communities. **Table C.** List of all variables used in the SEM analysis. Perceived vulnerability to leptospirosis was used as a binary variable (1–3 = not vulnerable, and 4–5 = vulnerable). **Fig D.** Summary of variables measured in the four communities (excluding those listed in main text Table 1). **Table E.** The top five models (lowest AIC score) for predicting leptospirosis sero-positivity in residents. The comparisons are among models with all possible combinations of candidate predictor variables. These predictors were variables that were maintained in the final structural equation model (see methods and results). All models included community identity (fixed effect) and household identity as a random effect.
(DOCX)
