# Supplementary material for: Poverty, sanitation, and Leptospira transmission pathways in residents from four Brazilian slums
Source: PLoS Negl Trop Dis. 2021 Mar 31;15(3):e0009256. doi: 10.1371/journal.pntd.0009256 (PMC8041187; doi:10.1371/journal.pntd.0009256)
Supplement: S1 File — Table A. Hypothesized relationships in the structural equation model (a) kept in the final model and b) that were removed (p > 0.1). The relationships that were removed included the effects of trash collection on rat sightings and trash accumulation, the relationship between sanitation and peri-domestic quality score and sero-positivity, and socioeconomic status score and exposure. The numbers next to the paths refer to the studies, listed below, that reported the hypothesized relationships. Table B. Full list of questions in the sero-survey applied in the four communities. Table C. List of all variables used in the SEM analysis. Perceived vulnerability to leptospirosis was used as a binary variable (1–3 = not vulnerable, and 4–5 = vulnerable). Fig D. Summary of variables measured in the four communities (excluding those listed in main text Table 1). Table E. The top five models (lowest AIC score) for predicting leptospirosis sero-positivity in residents. The comparisons are among models with all possible combinations of candidate predictor variables. These predictors were variables that were maintained in the final structural equation model (see methods and results). All models included community identity (fixed effect) and household identity as a random effect. (DOCX) [file pntd.0009256.s001.docx]

Supplementary information

**Table A**
Summary of variables measured in the four communities (excluding those listed in main text table 1)

|  | **Marechal Rondon** | **Alto do Cabrito** | **Nova Constituinte** | **Rio Sena** | **Four Communities** |
| --- | --- | --- | --- | --- | --- |
| Trash accumulates near the household (Trash piles) | % Yes | % Yes | % Yes | % Yes | % Yes |
|  | 45.6 | 38.1 | 30.1 | 28.4 | 36.0 |
| Rat sighting in the last 12 months | % Yes | % Yes | % Yes | % Yes | % Yes |
|  | 56.2 | 53.9 | 46.7 | 47.5 | 51.4 |
| Rodenticide application in household by the Center for Control of Zoonoses (CCZ) in past 12 months | % Yes | % Yes | % Yes | % Yes | % Yes |
|  | 54.4 | 74.1 | 87.9 | 78.9 | 73.4 |
| Access to household is paved | % Yes | % Yes | % Yes | % Yes | % Yes |
|  | 78.4 | 88.0 | 78.8 | 55.2 | 75.9 |
| House invaded by floodwater in the past 12 months | % Yes | % Yes | % Yes | % Yes | % Yes |
|  | 29.6 | 4.8 | 16.7 | 22.5 | 16.7 |
| The sewer close to the house floods when it rains (only those with an open sewer near the household answered) | % Yes | % Yes | % Yes | % Yes | % Yes |
|  | 43.8 | 15.7 | 24.2 | 22.7 | 26.5 |
| Own at least one automobile (head of the household responds) | % Yes | % Yes | % Yes | % Yes | % Yes |
|  | 5.0 | 6.1 | 3.2 | 3.0 | 4.5 |
| ***Continued below*** |  |  |  |  |  |

|  | | **Marechal Rondon** | | **Alto do Cabrito** | | | **Nova Constituinte** | | **Rio Sena** | **Four Communities** |
| --- | --- | --- | --- | --- | --- | --- | --- | --- | --- | --- |
| Own at least one laptop (head of household responds) | | % Yes | | % Yes | | | % Yes | | % Yes | % Yes |
|  |  | 8.0 | | 11.3 | | | 11.1 | | 8.3 | 9.7 |
| Own at least one automatic or semiautomatic washing machine (head of the household responds) | | % Yes | | % Yes | | | % Yes | | % Yes | % Yes |
|  |  | 22.5 | | 22.4 | | | 15.3 | | 15.0 | 19.1 |
| Running out of food in the house before they could afford more in the last 12 months (head of the household responds) | | % Yes | | % Yes | | | % Yes | | % Yes | % Yes |
|  |  | 49.1 | | 29.9 | | | 36.3 | | 40.1 | 38.6 |
| Walls are plastered (head of the household responds) | | % Yes | | % Yes | | | % Yes | | % Yes | % Yes |
|  |  | 11.8 | | 5.3 | | | 6.5 | | 25.1 | 11.8 |
| Education level | | % with less than 9 yrs education | | % with less than 9 yrs education | | | % with less than 9 yrs education | | % with less than 9 yrs education | % with less than 9 yrs education |
|  |  | 61.0 | | 51.6 | | | 58.8 | | 75.6 | 60.1 |
| Resident had contact with mud in the last 12 months | | % Yes | | % Yes | | | % Yes | | % Yes | % Yes |
|  |  | 44.4 | | 30.4 | | | 39.9 | | 34.4 | 37.1 |
| Resident had contact with floodwater in the last 12 months | | % Yes | | % Yes | | | % Yes | | % Yes | % Yes |
|  |  | 47.3 | | 27.7 | | | 41.2 | | 34.8 | 37.5 |
| ***Continued below*** | |  | |  | | |  | |  |  |
|  | | **Marechal Rondon** | | **Alto do Cabrito** | | | **Nova Constituinte** | | **Rio Sena** | **Four Communities** |
| Resident cleaned a blocked canal or sewer in the last 12 months | | % Yes | | % Yes | | | % Yes | | % Yes | % Yes |
|  |  | 12.7 | | 7.4 | | | 13.4 | | 13.7 | 10.8 |
| Resident cleans inside the house | | % Yes | | % Yes | | | % Yes | | % Yes | % Yes |
|  |  | 78.4 | | 76.0 | | | 87.6 | | 79.6 | 80.1 |
| Avoid trash accumulation inside the house | | % Yes | | % Yes | | | % Yes | | % Yes | % Yes |
|  |  | 45.3 | | 49.6 | | | 52.0 | | 53.8 | 50.0 |
| Resident kills rat in the household (traps and poison) | | % Yes | | % Yes | | | % Yes | | % Yes | % Yes |
|  |  | 22.5 | | 19.5 | | | 18.6 | | 23.4 | 20.9 |
| Resident cleans outside the house | | % Yes | | % Yes | | | % Yes | | % Yes | % Yes |
|  |  | 35.2 | | 29.3 | | | 47.7 | | 40.1 | 37.6 |
| Wear protective clothing outside the house (boots, gloves, closed shoes, etc) | | % Yes | | % Yes | | | % Yes | | % Yes | % Yes |
|  |  | 41.4 | | 37.3 | | | 21.9 | | 27.8 | 32.6 |
| Resident fixes or restructures sewers | | % Yes | | % Yes | | | % Yes | | % Yes | % Yes |
|  |  | 9.7 | | 7.7 | | | 7.2 | | 5.0 | 7.5 |
|  |  | |  | |  |  | |  |  |  |

**Table B**

Full list of questions in the sero-survey applied in the four communities.

| Variable | Possible responses |
| --- | --- |
| number of the house (as numbered by the team) |  |
| Initials of the interviewer |  |
| initials of the second member of the team |  |
| neighborhood | Marechal Rondon; Alto do Cabrito; Nova Constituinte; Rio Sena |
| house block |  |
| number of the house within block |  |
| usage of the domicile | empty; abandoned; construction site; commerce; residence; residence and commerce |
| participation | accepted; refused; not found; no access |
| number of inhabitands |  |
| phone 1 |  |
| phone 2 |  |
| phone 3 |  |
| are there public lights in the street? | Y/N |
| are there open sewers near the house? | Y/N |
| does the nearest stretch of sewers close to your home present any anti-flooding physical barriers? | Y/N |
| how does the inhabitant prevents floodwaters from invading their home? | walls; raised door stoop in the backyard; raised door stoop in the entrance; raised house floor; occupation of a higher story; none |
| does the access road is paved? | Y/N |
| is the house built in a slope? | Y/N |
| predominant material of the house walls | concrete or plastered bricks; unplastered bricks; wood or other not brick-and-mortar materials |
| backyard | Y/N |
| backyard floor | unpaved; concrete; tiles |
| house with freshwater plumming | Y/N |
| house with the water company's consumption counter | Y/N |
| water shortage wthin the last 30 days | Y/N |
| number of days without water last month |  |
| does the house has water storages? | Y/N |
| use of barrels for water storage | yes; no; not sure |
| water tank | yes; no; not sure |
| cistern | yes; no; not sure |
| drums | yes; no; not sure |
| does your house had spots where rainwater accumulated in the last 30 days? | Y/N |
| lake | yes; no; not sure |
| potted plants | yes; no; not sure |
| small recipients (cups, bottles, buckets and tins) | yes; no; not sure |
| foodstuff storage | plastic bags in a cupboard; only plastic bags; other |
| (follow up) other |  |
| within 10 meters, is there a junkyard/recycling plant/garbage dump? | Y/N |
| is there trash collection in your street? | Y/N |
| final destination of domestic trash | burnt; buried; open air dump; other |
| (follow up) other |  |
| how do you discard your trash | only plastic bags; only containers with lids; both; other |
| (follow up) other |  |
| where do you deposit your trash for pickup? | directly in front of the house; suspended; collective container; empty lot; other |
| (follow up) other |  |
| frequency of trash collection | daily; once a week; twice a week; thrice a week |
| animals in the household | Y/N |
| species | cat;dog;chicken;other |
| number of cats |  |
| number of dogs |  |
| number of chicken |  |
| other animals |  |
| how long since the last rodent control activity from CCZ in your house/street? | six months; over an year; never |
| type of control method deployed by the CCZ | contact powder; blocks; don't know |
| how long since the health agents visited your house? | six months; over an year; never |
| number of heath agent visits in the last six months |  |
|  |  |
| **head of household** |  |
| name |  |
| income |  |
| name of inhabitant 2 |  |
| income of inhabitant 2 |  |
| (repeat to 10) |  |
|  |  |
| **individual** |  |
| initials of interviewer |  |
| number of the person within household |  |
| IDIND |  |
| RG (ID) |  |
| full name |  |
| social name |  |
| sex |  |
| birthday |  |
| age |  |
| calculated age |  |
| consent | Y/N |
| date of consent check |  |
| reason | refusal; moved; not found; dead; other |
| definition of refusal | partial; final |
| not found | confirmed by resident; not confirmed |
| blood sample collected | Y/N |
| reason for no collection |  |
| date of collection |  |
| interview method | online; offline |
| interviewer initials |  |
| phone number |  |
| additional phone number |  |
| civil state | single; lawfully wed; stable union (6+ months); widow; divorced or separated |
| self-defined race | white; black; yellow; brown, indigenous |
| do you currently study, or intend to? | currently studies; intends to; do not intend to |
| last school year coursed | never studied; alphabetization school (1st year); 1st grade; 2nd grade; 3rd grade; grade 4; grade 5; grade 6; grade 7; grade 8; grade 9; high school junior; high school sophomore; high school veteran; incomplete university degree; university degree |
| employment | Y/N |
| employment type | formal; informal |
| does your occupation involve: | construction work; street vendor; sanitation and recycling services; works in contact with mud; works in com tact with floodwater; works in contact with sewage; none |
| income |  |
| have you entered in contact with mud in the last 12 months? | Y/N |
| frequency in the last 12 months | rare; seldom; frequent |
| frequency of use of protective boots when in contact with mud | rare; seldom; frequent; never |
| have you entered in contact with floodwater in the last 12 months? | Y/N |
| frequency in the last 12 months | rare; seldom; frequent |
| frequency of use of protective boots when in contact with floodwater | rare; seldom; frequent; never |
| have you entered in contact with sewage in the last 12 months? | Y/N |
| frequency in the last 12 months | rare; seldom; frequent |
| frequency of use of protective boots when in contact with sewage | rare; seldom; frequent; never |
| have tiy walked barefeet outside your home in the last 12 months | Y/N |
| frequency in the last 12 months | rare; seldom; frequent |
| type of shoe normally used | open; closed |
| have you worked in unclogging a sewer canal in the last 12 months? | Y/N |
| if so, the frequency of protective boot use | rare; seldom; frequent; never |
| how serious do you think leptospirosis is | scale of 1-10 |
| how vulnerable to leptospirosis contamination do you believe you are | scale of 1-5 |
| what are your main worries in your community | violence; health; finances; other |
| (follow up) other |  |
| do you know leptospirosis | Y/N |
| if so, how did you heard of it | family; acquaintances; work; health services; media; school |
| how does leptospirosis contamination happes? | contact with rat urine; contact with rat feces; contact/presence of rats (w/o mentioning urine/feces); walking barefoot; contact with mud, contact/rpresence of trash; when it rains; other |
| (follow up) other |  |
| how do you avoid contamination? | inside the house: cleaning; inside the house: not accumulating trash; inside the house: walking with shoes; inside the house: kill rats; outside the house: cleaning; outside the house: using protection (boots, shoes, gloves); outside the house: kill rats; outside the house: fix/improve sewers; outside the house: not accumulating trash; other |
| (follow up) other |  |
| what would be more important to recude the risk of contracting leptospirosis in your opinion? | State interventions (closing sewers, trash collection, unclogging canals, zoonosis control, health services initiatives); personal action; action amongs your neighbors |
| which State actions? | closing sewers; trash collection; unclogging canals; zoonosis control; health services initiatives |
| who is the one responsible for this aspect on the family? |  |
| how long have you lived here? |  |
| how many people sleep at least 3 nights/week in the past month? |  |
| is the house owned or rented? |  |
| do you have the legal documents of the house |  |
| have you at any point in the last 12 months feared your house would run out of food before you could buy or receive more? | never; sometimes; frequently |
| in the last 12 months, have you ran out of food before you could afford more | never; sometimes; frequently |
| in the last 12 months, did any floodwater invade your home | Y/N |
| in the last 30 days, have you seen rats close (<10m) to your home | Y/N |
| what was the most rats you've seen at once? |  |
| in the last 30 days, have you seen any trash pilling up near (<10m) your home (besides your own) |  |
| does the sewers close to your home flood when it rains? | Y/N |
| are landslides a problem in your neighborhood? | Y/N |
| did a landslide occurred close to your home in the last 6 months | Y/N |
| does your family receive Bolsa família? | Y/N |
| if so, how much? |  |
| how many TV sets? |  |
| number of restrooms exclusive to the household |  |
| number of restrooms without a toilet exclusive to the household |  |
| number of VCR/DVD sets |  |
| number of refrigeratos |  |
| number of freezers |  |
| number of washing machines |  |
| number of computers |  |
| number of radio sets |  |
| number of microwave ovens |  |
| number of automobiles |  |
| number of motorcicles |  |
| access to the internet | Y/N |
| type of access | mobile; LAN/WAN |
| difficulty to access the main road in the neighborhood? | Y/N |
| why the difficulty of accessing the main road | reduced mobility; floods; topografy; other |
| (follow up) other |  |
| do you use protective boots when needed | yes, my own; yes, I can borrow; no |
| how many mornings did you spent in this house last week |  |
| how many afternoons did you spent in this house last week |  |

**Table C**

List of all variables used in the SEM analysis. Perceived vulnerability to leptospirosis was used as a binary variable (1-3 = not vulnerable, and 4-5 = vulnerable).

| Variable groups | Type of variable |
| --- | --- |
| **Peri-domestic environment** |  |
| whether the access to the house is paved or not | Yes/no |
| was the house invaded by floodwater in the past 12 months? | Yes/no |
| does the sewer close to your house floods when it rains? | Yes/no |
| is there an open sewer close to your house? | Yes/no |
| is there a junkyard or garbage pile within 10m of the household? | Yes/no |
| in the past 30 days, have you seen a rat within 10m of the household? | Yes/no |
| **Socioeconomic status** |  |
| do you own at least one automobile? (head of the household) | Yes/no |
| do you own at least one computer? (head of the household) | Yes/no |
| do you own at least one automatic or semiautomatic washing machine? (head of the household) | Yes/no |
| in the past 12 months, have you been at risk of running out of food in the house before you could afford more? (head of the household) | Yes/no |
|  |  |
| is the house owned or rented (head of the household) | Owned/rented |
| maximum education attained | Never studied, <5 years, 5-9 years , >9 years |
| maximum income in $R in the household (head of the household) | Integer |
| walls are plastered?* (head of the household) | Plastered/unplastered |
| **Exposure to sources of contamination** |  |
| in the past 12 months, have you walked through or got in contact with sewers? | Yes/no |
| in the past 12 months, have you got in contact with flooding water? | Yes/no |
| in the past 12 months, have you cleaned and/or unclogged a sewer canal? | Yes/no |
| in the past 12 months, have you walked through or got in contact with mud? | Yes/no |
| **Individual protective measures** |  |
| inside the house: cleaning the house | Yes/no |
| inside the house: avoid trash accumulation | Yes/no |
| inside the house: killing rats | Yes/no |
| outside the house: cleaning | Yes/no |
| outside the house: using protection | Yes/no |
| outside the house: killing rats | Yes/no |
| outside the house: fix or restructure the sewers | Yes/no |
| **Ongoing interventions** |  |
| is there garbage collection in your street? | Yes/no |
| in the past 6 months, have you received a visit from CCZ agents? | Yes/no |
| **Individual variables** |  |
| how vulnerable to leptospirosis do you think you are? | Scale (1 -5) |
| gender | female/male |
| age (calculated) | Integer |
| * Unplastered walls often have holes in them and are more likely to leak water |  |

**Fig D**

Hypothesized relationships in the structural equation model (**a)** kept in the final model and **b)** that were removed (*p* > 0.1). The relationships that were removed included the effects of trash collection on rat sightings and trash accumulation, the relationship between sanitation and peri-domestic quality score and sero-positivity, and socioeconomic status score and exposure. The numbers next to the paths refer to the studies, listed below, that reported the hypothesized relationships.


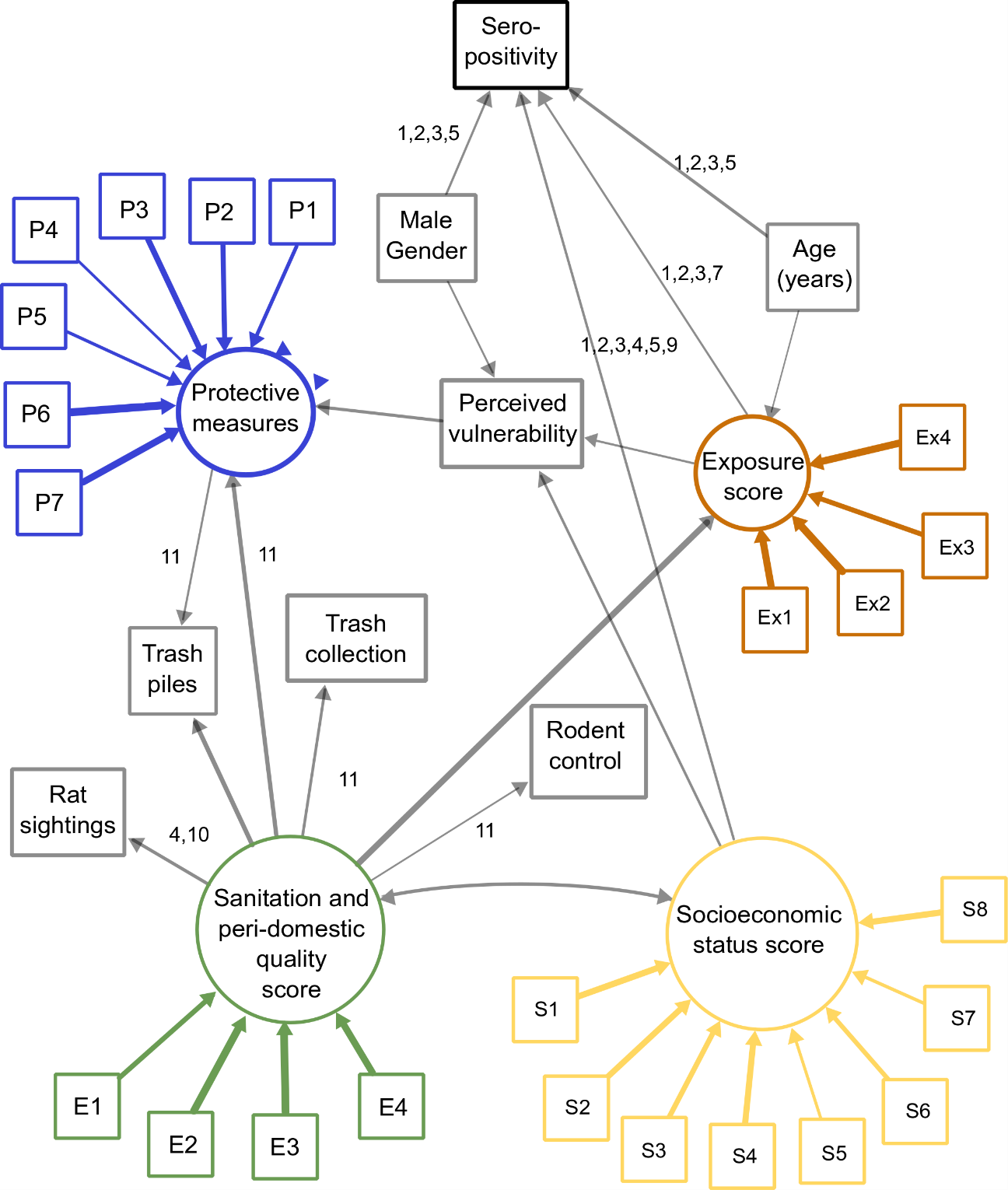


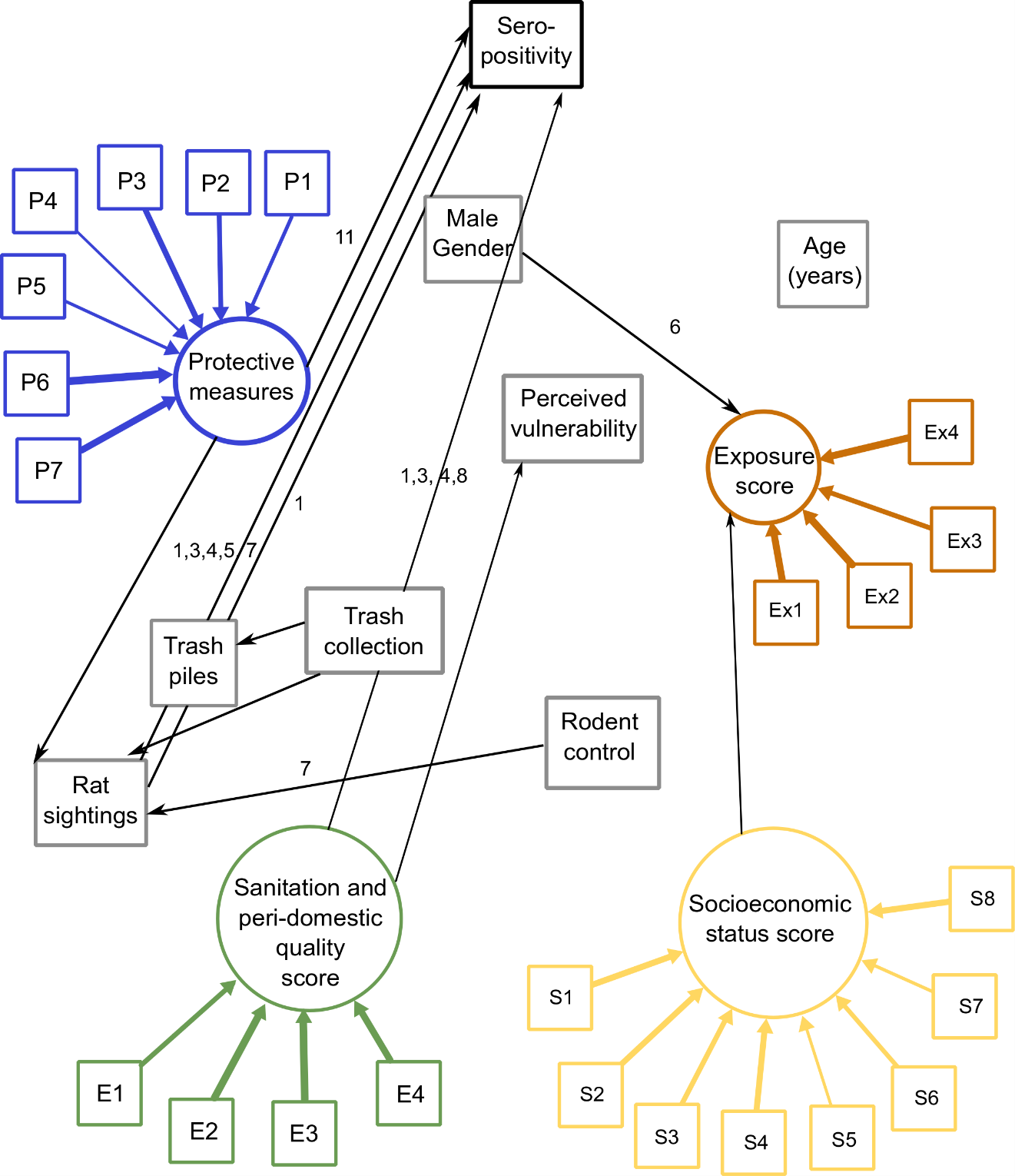


1 – Reis RB, Ribeiro GS, Felzemburgh RD, et al. Impact of environment and social gradient on Leptospira infection in urban slums. *PLoS Negl Trop Dis* 2008; **2**(4): e228.

2 – Felzemburgh RD, Ribeiro GS, Costa F, et al. Prospective study of leptospirosis transmission in an urban slum community: role of poor environment in repeated exposures to the Leptospira agent. *PLoS Negl Trop Dis* 2014; **8**(5): e2927

3 – Hagan JE, Moraga P, Costa F, et al. Spatiotemporal Determinants of Urban Leptospirosis Transmission: Four-Year Prospective Cohort Study of Slum Residents in Brazil. *PLoS Negl Trop Dis* 2016; **10**(1): e0004275

4 – Costa F, Ribeiro GS, Felzemburgh RD, et al. Influence of household rat infestation on leptospira transmission in the urban slum environment. *PLoS Negl Trop Dis* 2014; **8**(12): e3338.

5 – Ko AI, Reis MG, Dourado CMR, Johnson WD, Riley LW, Grp SLS. Urban epidemic of severe leptospirosis in Brazil. *Lancet* 1999; **354**(9181): 820-5.

6 – Navegantes de Araújo W, Finkmoore B, Ribeiro GS, et al. Knowledge, Attitudes, and Practices Related to Leptospirosis among Urban Slum Residents in Brazil. *The American Journal of Tropical Medicine and Hygiene* 2013; **88**(2): 359-63.

7 – Richardson JL, Silveira G, Soto Medrano I, et al. Significant Genetic Impacts Accompany an Urban Rat Control Campaign in Salvador, Brazil. *Frontiers in Ecology and Evolution* 2019; **7**(115).

8 – Sarkar U, Nascimento SF, Barbosa R, et al. Population-based case-control investigation of risk factors for leptospirosis during an urban epidemic. *Am J Trop Med Hyg* 2002; **66**(5): 605-10.

9 – Kikuti M, Cunha GM, Paploski IAD, et al. Spatial Distribution of Dengue in a Brazilian Urban Slum Setting: Role of Socioeconomic Gradient in Disease Risk. *Plos Neglect Trop D* 2015; **9**(7). (**Dengue fever**)

10 – Bonner PC, Schmidt WP, Belmain SR, Oshin B, Baglole D, Borchert M. Poor housing quality increases risk of rodent infestation and Lassa fever in refugee camps of Sierra Leone. *Am J Trop Med Hyg* 2007; **77**(1): 169-75 (**Lassa fever**)

11 – Corburn J, Riley LEE. From the Cell to the Street Coproducing Slum Health. In: Corburn J, Riley L, eds. Slum Health. 1 ed: University of California Press; 2016: 11-37 (**Slum health**)

**Table E**

Comparisons of generalized linear mixed effect models (GLMMs) with binomial error distribution predicting sero-positivity status in residents. In all models, household identity was added as a random effect and community identity (Marechal Rondon, Alto do Cabrito, Nova Constituinte, and Rio Sena) was included as a fixed effect in all competing models (not shown among variables below). Thus, all models had community and household effects controlled for, and comparisons were only based on the variables listed below. Model 1, with the lowest AIC, was selected as the final model.

| Model ranking | Variables | AIC | ΔAIC |
| --- | --- | --- | --- |
| **1** | Socioeconomic score + Exposure score + Sex + Age | 649.9 | 0 |
| 2 | Socioeconomic score + Sex + Age | 653.5 | 3.6 |
| 3 | Socioeconomic score + Exposure score + Age | 656.8 | 6.9 |
| 4 | Socioeconomic score + Sex + Age | 657.4 | 7.5 |
| 5 | Socioeconomic score + Age | 664.6 | 14.7 |
